# Ultrasonographic Assessment of Enthesitis in HLA-B27 Positive Patients with Rheumatoid Arthritis, a Matched Case-Only Study

**DOI:** 10.1371/journal.pone.0058616

**Published:** 2013-03-07

**Authors:** Antonio Mera-Varela, Aida Ferreiro-Iglesias, Eva Perez-Pampin, Marisol Porto-Silva, Juan J. Gómez-Reino, Antonio Gonzalez

**Affiliations:** 1 Research Laboratory 10 and Rheumatology Unit, Instituto de Investigacion Sanitaria – Hospital Clinico Universitario de Santiago, Santiago de Compostela, Spain; 2 Department of Medicine, University of Santiago de Compostela, Santiago de Compostela, Spain; University of Texas Southwestern Medical Center, United States of America

## Abstract

**Introduction:**

HLA-B27 has a modifier effect on the phenotype of multiple diseases, both associated and non-associated with it. Among these effects, an increased frequency of clinical enthesitis in patients with Rheumatoid Arthritis (RA) has been reported but never explored again. We aimed to replicate this study with a sensitive and quantitative assessment of enthesitis by using standardized ultrasonography (US).

**Methods:**

The Madrid Sonography Enthesitis Index (MASEI) was applied to the US assessment of 41 HLA-B27 positive and 41 matched HLA-B27 negative patients with longstanding RA. Clinical characteristics including explorations aimed to evaluate spondyloarthrtitis and laboratory tests were also done.

**Results:**

A significant degree of abnormalities in the entheses of the patients with RA were found, but the MASEI values, and each of its components including the Doppler signal, were similar in HLA-B27 positive and negative patients. An increase of the MASEI scores with age was identified. Differences in two clinical features were found: a lower prevalence of rheumatoid factor and a more common story of low back pain in the HLA-B27 positive patients than in the negative. The latter was accompanied by radiographic sacroiliitis in two HLA-B27 positive patients. No other differences were detected.

**Conclusion:**

We have found that HLA-B27 positive patients with RA do not have more enthesitis as assessed with US than the patients lacking this HLA allele. However, HLA-B27 could be shaping the RA phenotype towards RF seronegativity and axial involvement.

## Introduction

Research in the genetic component of RA etiology has experienced a marked progress in recent years with the identification of a large number of susceptibility loci [1], [2]. However, there are still many unsolved questions. Among them the relative to genotype-phenotype relationships are very prominent. It is expected that genotypes will have an important role in shaping the RA phenotype and that identification of these relationships will help manage patients in a personalized manner. The most established relationship has been the observed between a large number of RA susceptibility factors and the production of anti-citrullinated protein antibodies (ACPA). The earlier discovered association of the shared epitope (SE) with severe RA, including progression of erosive arthritis, seems to be explained by SE association with ACPA production. Other lines of active research in this field include the search for genetic factors modifying progression of erosions [3], [4], or response to treatment [5], [6] and of loci associated with ACPA negative patients [7], [8]. Some of these studies are focused in RA susceptibility loci [4], [6], [8], which are good candidates because of their involvement in the disease mechanisms. Additional candidates are the genetic factors associated with related diseases. These loci are also of known functional relevance and they have the potential of being involved in subgroups of RA patients sharing clinical characteristics with the disease where the loci were identified.

One of these candidates is HLA-B27 [9], [10], [11], [12], [13]. It is strongly associated with ankylosing spondylitis (AS) and with other spondyloarthritis (SpA), but not with RA [14], [15]. HLA-B27 has also modifier effects on the phenotype that have been observed in its associated diseases and in some non-HLA-B27 associated diseases. Among the HLA-B27 associated diseases, there are multiple reports showing earlier disease onset, more involvement of sacroiliac and spine joints, a higher tendency to chronicity and more prevalence of back pain in HLA-B27 positive than in negative patients [16], [17], [18], [19], [20], [21]. In addition, non-HLA-B27 associated diseases can be modified to show higher prevalence of archetypical AS clinical features. For example, HLA-B27 positive patients with inflammatory bowel disease show an increased prevalence of sacroiliitis, spondylitis and enthesitis [22], [23]. HLA-B27 also increases the prevalence of sacroiliitis in patients with familial Mediterranean fever [24]; and of sacroiliitis, inflammatory back pain and enthesitis in children with Juvenile Idiopatic Arthritis [25], [26]. Therefore, it is not surprising that some researchers have thought the presence of HLA-B27 could modify the phenotypes of patients with RA towards resembling SpA. There have been some reports in this direction [9], [10], [11], [12], [13], but all these studies were done more than a decade ago and none of their findings have become established. One of these studies reported an increased prevalence of clinical enthesitis in patients with early arthritis fulfilling RA criteria [9].

Enthesitis, the inflammation of the entheses, is very prevalent and relatively specific of all forms of SpA motivating its inclusion as part of the disease classification criteria [15], [27]. Enthesitis is present in early SpA and, occasionally, it is the unique disease manifestation for some time. This has lead some authors to consider enthesitis the primary lesion in this group of diseases [28]. Now we have the possibility to identify early enthesitis when it is not yet clinically apparent either to patients or to physical exploration thanks to magnetic resonance imaging and ultrasonography (US) [29], [30], [31], [32], [33]. This property of US has been applied to a variety of clinical situations demanding sensitive evaluation. Examples include SpA with subclinical enthesopathy [34], recurrent acute anterior uveitis without SpA [35], long term dialysis [36], and patients with psoriasis without psoriatic arthritis [36], [37], showing in all these cases an increased frequency of abnormalities. US evaluation has also shown an increased frequency of abnormalities in patients with RA relative to healthy controls in spite of the complete absence of clinical enthesitis [38], [39]. Only one of these studies assessed HLA-B27, concerning recurrent anterior uveitis, and it found that enthesitis was associated with this HLA allele [35].

Therefore, we have now the opportunity to analyze the prevalence of enthesitis in RA patients stratified for HLA-B27 status with the sensitivity and accuracy allowed by US. With this aim, we have selected all available RA patients positive for HLA-B27 in our hospital and matched RA patients negative for HLA-B27. The two groups were compared with the Madrid Sonography Enthesitis Index (MASEI) [31], focused anamnesis and physical exploration. No differences in the MASEI or any of its components were detected. This result excludes a role of HLA-B27 in the enthesal abnormalities of these patients. However, a modifier effect of HLA-B27 on the phenotype of the patients with RA was suggested by its association with RF negative status and a more frequent story of low back pain.

## Materials and Methods

### Ethics statement

All patients gave their written informed consent to participate. Sample collection and the study protocol were approved by the Comite de Investigacion Clinica de Galicia (Spain).

### Selection of patients

DNA and serum samples from patients with RA according to ACR classification criteria were used [40]. All patients were of Spanish ancestry and were attending the Rheumatology Unit of the Hospital Clinico Universitario de Santiago. HLA-B27 positive patients were selected. Gender, age at disease onset and current age of these patients was used to select matched HLA-B27 negative patients at a 1∶1 ratio. Patients were invited to participate in the study and they gave their written informed consent. The rheumatologist and the nurse involved in recruitment and evaluation of the patients were blind to their HLA-B27 status.

### Laboratory studies

Two complementary genotyping reactions were used to determine the HLA-B27 status (available from the authors upon request). The first PCR was based on the method of Bon et al. [41]. It amplifies HLA-B*2701-*2706 alleles, which are the most common in the Caucasian population and the most associated with AS. The second PCR used a combination of 3 primers described by Faner et al. [42]. It amplifies a larger number of alleles than the first, from HLA-B*2701 to *2724 except for HLA-B*2718 and HLA-B*2723. PCR amplification was done with a multiplex PCR system (KAPA2G fast HotStart, Kapa Biosystems, Woburn, MA). Detection of the PCR products was performed with single-base extension (SNaPshot Multiplex Kit from Applied Biosystems, Foster City, CA) using probes targeting a non polymorphic base. In this way, the most frequents alleles of HLA-B27 were covered by two PCR. Four samples were positive only in the second amplification, which is consistent with the low frequency of the rare alleles in Spain [43].They were excluded from analysis because of their more uncertain status.

HLA-DBR1 alleles were determined by a sequencing based typing method (SBT) using the AlleleSEQR HLA-DRB1 Typing kit (Abbott Diagnostics, Abbott Park, Germany), which includes bidirectional sequencing of the second exon of DRB1. Ambiguous samples were additionally sequenced with group-specific primers (AlleleSEQR HLA-DRB1 GSSP, Abbott). The anti-CCP status of the patients was determined using the EDIA ACPA Kit (Euro-Diagnostica, Arnhem, The Netherlands). Quantification and setting of the cut-off level at 5 units/ml were done according to the manufacturer's instructions. RF status was determined by rate nephelometry with the IMMAGE Immunochemistry Systems (Beckman Coulter, Ireland), which covers all Ig isotypes.

### Clinical and Ultrasound evaluation

Patient history was reviewed and a specific anamnesis of symptoms and signs characteristic of HLA-B27 associated diseases was conducted. Physical exploration was done looking for signs of axial involvement with instruments developed for SpA as the Schober test, BASDAI and BASFI scores and assessment of the lateral trunk flexion. A modified Schober test according with Moll and Wright was used[44]. Inflammatory back pain was defined as lumbar pain at night or with morning stiffness that does not improve with rest or that improves with exercise and that persists for more than a month. Plain antero-posterior pelvic radiographs were evaluated for the presence of sacroiliitis. This assessment was independently done by two rheumatologists in a blind form.

In addition, the presence of enthesitis was evaluated by a rheumatologist experienced in US focused on articular and periarticular locations and blind to the HLA-B27 status of patients. A General Electric LogiqQ7 US machine with a 10–14 MHz linear array transducer was used. The power Doppler setting was standardized with a pulse repetition frequency (PRF) of 500 Hz with a low wall filter and 35–40 dB of gain. The Madrid Sonography Enthesitis Index (MASEI) was used for quantitative and standardized assessment [31]. This index evaluates 6 features in 6 entheses. A value ≥ 18 has been defined as characteristic of SpA. The 6 features are enthesis thickness, structure, calcifications, erosions, bursae and power Doppler signal. The 6 bilaterally assessed locations are proximal plantar fascia, distal Achilles tendon, distal and proximal patellar tendon insertion, distal quadriceps tendon and brachial triceps tendon.

### Statistical analysis

HLA-B27 positive patients with RA were considered cases and the matched HLA-B27 negative patients were controls. Demographic, clinical, laboratory, radiographic and ultrasound characteristics were compared between the two groups using Student T test or the Fisher exact test for contingency tables depending on the quantitative or qualitative nature of the variables, respectively. Detailed US results were compared with the Man-Whitney U test and non-parametric statistics because they showed many zero values. All analyses were done with Statistica 7.0 (StatSoft, Tulsa, OK). Differences with P<0.05 were considered statistically significant.

## Results

Analysis of 672 patients with RA identified 65 that were HLA-B27+, the remaining 607 were HLA-B27- (11 additional patients showed an uncertain HLA-B27 status and were not included). The HLA-B27 positive subgroup of patients showed less RF positive subjects, 50.0 %, than the HLA-B27 negative subgroup 64.6 % (P = 0.025). Also the ACPA status showed a trend to decreased prevalence in this subgroup (Table 1). However, there were not more patients with low titers of ACPA (between 5 and 45 units) in the HLA-B27 positive (33.3 %) than in the HLA-B27 negative subgroup (32.8 %). The other characteristics, gender, age at disease onset, erosive arthritis and carrier status of the SE, were similar in the two subgroups of patients (Table 1).

**Table 1:** Characteristics of the patients with RA in function of the HLA-B27 subgroup.

	HLA-B27-	HLA-B27+	*P* value
Women %	77.6 (471/607)^a^	72.3 (47/65)	0.3
Age of disease onset, mean (SD)	46.3 (14.8)	44.9 (16.7)	0.5
Rheumatoid Factor % b	64.6 (369/571)	50.0 (30/60)	0.025
Anti-CCP %	63.0 (376/597)	50.8 (33/65)	0.05
Anti-CCP median (IQR) c	93.3 (32.8−198.0)	67.4 (18.7−141.3)	0.3
Carrier SE %	53.3 (286/537)	56.4 (31/55)	0.7
Erosive arthritis %	65.8 (369/561)	61.7 (37/60)	0.5

a Number with the feature/total number of patients with available information.

b Median and IQR were 122 (61−445) in the HLA-B27- subgroup and 235 (128−366) in the HLA-B27+ subgroup (*P* = 0.8). This information was available only for 127 RF+ patients.

c Median and interquartile range of the anti-CCP positive patients.

A total of 41 HLA-B27 positive patients were available and willing to participate in the study. One HLA-B27- patient was selected for each HLA-B27+ patient trying to match them for gender, age at disease onset and current age. The resulting groups were very similar not only in the selected variables but also in other characteristics as height, weight, serology and SE status (Table 2). During the visit for evaluation one of the patients in the HLA-B27 positive subgroup was found to have SpA in spite of his previous classification as RA and was excluded from further analysis. Specific anamnesis and physical evaluation of the patients were done blind to their HLA-B27 status. They disclosed a higher prevalence of low back pain, mechanical or inflammatory, in the HLA-B27 positive subgroup, 27.5 %, than in the HLA-B27 negative subgroup, 7.5 %, P = 0.037. This difference was similarly distributed between inflammatory and mechanical pain (Table 3). No other symptom or evaluation showed differences between the two patient groups. Specifically there were not differences in modified Schober's test, BASDAI or BASFI scores or in the lateral flexion of the spine (Table 3). A few patients reported a story of past skin lesions that could correspond to psoriasis but without differences between the two subgroups.

**Table 2:** Characteristics of the patients recruited for detailed analysis in function of the HLA-B27 subgroup.

	HLA-B27-	HLA-B27+	*P* value
Women %	73.2 (30/41)^a^	80.5 (33/41)	0.6
Age of disease onset, mean (SD)	41.9 (15.7)	43.9 (17.3)	0.6
Current age, mean (SD)	64.8 (15.2)	64.4 (14.7)	0.9
Weight, mean (SD)	68.8 (12.2)	68.3 (13.7)	0.8
Height, mean (SD)	159.5 (7.8)	158.3 (8.7)	0.5
Ever smoking %	24.4 (10/41)	24.4 (10/41)	1
Rheumatoid factor %	55.0 (22/40)	50.0 (20/40)	0.8
Anti-CCP %	40.0 (16/40)	51.2 (21/41)	0.4
Carrier of SE %	55.0 (22/40)	57.9 (22/38)	0.8
Erosive arthritis %	65.0 (26/40)	53.9 (21/39)	0.4
Biologics %	50.0 (20/40)	60.0 (24/40)	0.5
Anti-TNF b	60.0 (12/20)	45.8 (11/24)	0.4
Rituximab	15.0 (3/20)	16.7 (4/24)	1.0
Abatacept	10.0 (2/20)	20.8 (5/24)	0.4
Tocilizumab	15.0 (3/20)	16.7 (4/24)	1.0
Methotrexate	65.0 (13/20)	83.3 (20/24)	0.2
Leflunomide	20.0 (4/20)	0.0 (0/24)	0.04

a Number with the feature/total number of patients with available information.

b Including Etanercept, Adalimumab and Infliximab.

**Table 3:** Results of the anamnesis and exploration of recruited patients in function of the HLA-B27 subgroup.

	HLA-B27-	HLA-B27+	*P* value
Back pain %	7.5 (3/40)	27.5 (11/40)	0.037
Inflammatory back pain %	2.5 (1/40)	12.5 (5/40)	0.2
Non-inflammatory back pain %	5.0 (2/40)	15.5(6/40)	0. 3
Story of ‘psoriatic’ lesions %	7.3 (3/40)	5.0 (2/40)	1.0
Schober's test, mean (SD)	3.7 (1.0)	3.8 (1.0)	0.9
Right lateral flexion, mean (SD)	13.2 (4.0)	12.7 (4.4)	0.6
Left lateral flexion, mean (SD)	13.4 (4.3)	11.8 (3.6)	0.1
BASDAI, mean (SD)	4.3 (2.5)	4.6 (2.4)	0.6
BASFI, mean (SD)	2.9 (2.4)	3.4 (2.2)	0.4
Sacroiliitis Rx %	0.0 (0/34)^a^	5.7 (2/35)	0.3

a Number with the feature/total number of patients with available information.

Two rheumatologists that were blind to the HLA-B27 status of the patients evaluated plain pelvic radiographs for the presence of sacroiliitis. Their assessment was fully concordant identifying 2 patients with sacroiliitis. The two were positive for HLA-B27. This was not significantly different from the result in the HLA-B27 negative subgroup, but we investigated it further. A review of the two patients with sacroiliitis did not challenge their classification as RA: the two showed erosive RA and one of them was positive for ACPA and homozygous for the SE. The two patients had a story of inflammatory low back pain.

**Figure 1:**
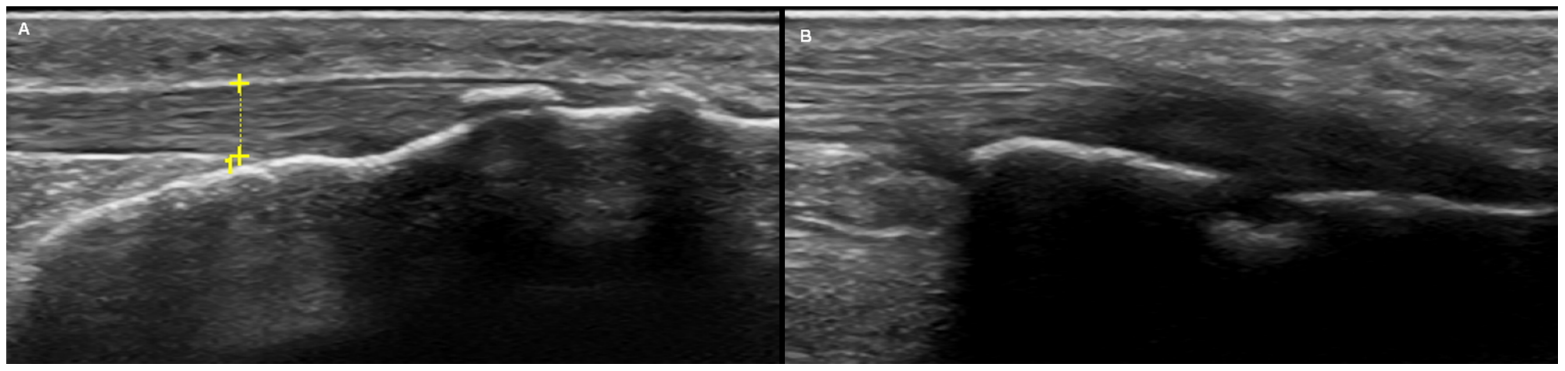
Representative images of features detected with US exploration. A) Example of analysis of the Achilles tendon thickness measured between the two yellow crosses in one patient, and B) Erosion detected in the superior pole of the calcaneous in a different patient.

**Figure 2:**
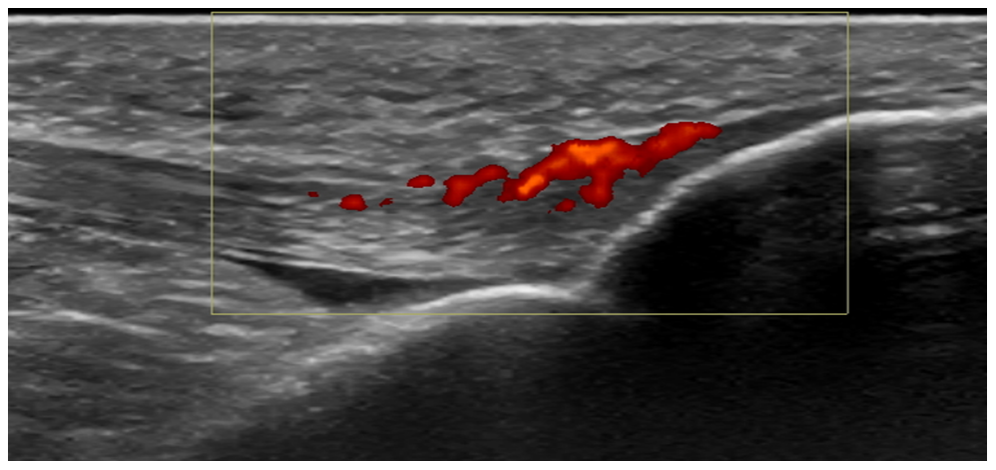
Positive power Doppler signal identifying tibial tuberosity enthesitis. The signal (in red) is detected in the tibial insertion of the patellar ligament in one of the studied patients.

Systematic evaluation of the entheses following the MASEI procedure (representative images in Figure 1) yielded no differences between the two subgroups of patients (Table 4). Mean MASEI values were even nominally lower in the HLA-B27 positive subgroup. Also, the threshold score of 18 proposed as specific of AS [31], was not discriminating between the two subgroups: there were 13 patients above this value in the HLA-B27 positive subgroup and 10 in the HLA-B27 negative subgroup. In addition, no difference was detected with any of the components of the index (Table 4). This is specially relevant for the power Doppler signal (Figure 2) because it has been differentially associated with SpA relative to other diseases including RA [39]. But no difference was detected comparing the whole distribution of values (Table 4), or the percentage of positive signals (9/40 in HLA-B-27 positive patients *vs*. 13/41 in HLA-B27 negative patients) or the mean values in the patients showing Doppler signals (4.7 *vs*. 4.6, in the HLA-B27 positive and negative patients, respectively). The only associations we identified were unrelated with the HLA-B27 status: a significant increase of the MASEI value in men relative to women (not shown), and an increase of the score with age due to calcifications (not shown). The two patients with sacroiliitis lacked signs of enthesitis (MASEI values of 4 and 8).

**Table 4:** Ultrasonographic evaluation of the entheses following the MASEI procedure.

	HLA-B27-	HLA-B27+	*P* value
MASEI, mean (SD)	14.2 (9.3)	13.6 (8.8)	0.7
MASEI, median (_10–90_R)*	11.0 (5−24)	11.5 (4−27)	0.7
MASEI >18, %	24.4 (10/41)	32.5 (13/40)	0.5
- structure, median (_10–90_R)	0 (0−1)	0 (0−1.5)	0.8
- thickness, median (_10–90_R)	2 (0−5)	2 (0−5)	0.08
- erosion, median (_10–90_R)	3 (0−6)	0 (0−10.5)	0.8
- calcification, median (_10–90_R)	7 (2−13)	5 (2.5−14)	0.7
- Doppler, median (_10–90_R)	0 (0−6)	0 (0−3)	0.3
- bursitis, median (_10–90_R)	0 (0−1)	0 (0−1)	0.8

* The range between percentiles 10 and 90.

## Discussion

Our analyses have not shown any specific enthesal abnormality in the HLA-B27 positive patients with RA. The sensitive and quantitative evaluation allows us to exclude a significant HLA-B27 modifier effect towards enthesitis in the RA patients. However, an excess of patients in the HLA-B27 positive group referred a story of low back pain that in two of the patients was associated with radiographic sacroiliitis. These findings together with a lower prevalence of RF keep open the possibility of a modifier effect of the HLA-B27 allele on the phenotype of RA patients, but excluding the presence of enthesitis.

Our primary aim was to compare the frequency of enthesitis between HLA-B27 positive and negative patients with RA using systematic US examination. This imaging technology has shown sensitivity for detecting a high percentage of SpA patients with subclinical enthesitis [15], [29], [30], [31], [32], [33], [34], [39]. It is applied with scoring protocols that help differentiate SpA patients from controls, from subjects that have suffered mechanical injury and from other forms of inflammatory arthritis [31], [32], [33]. Specifically, the MASEI protocol used here has shown high sensitivity and specificity and it is very comprehensive because it includes assessment of 6 features in 6 enthesal sites and both grey scales and power Doppler [31], [33]. The assessment of power Doppler is a distinct advantage of this method over the most commonly used GUESS procedure for our study because the Doppler signal is the most specific feature distinguishing SpA from mechanical and RA enthesal abnormalities [39].

We have found significant abnormalities in the entheses of patients with RA confirming findings of previous US studies [38], [39], [45]. However, no differences were found between HLA-B27 positive and negative patients, even for components of the index that are more specific for SpA like the Doppler signal. Therefore, it is clear that the presence of HLA-B27 does not induce enthesal pathology in RA patients and that all the abnormalities found in these patients are produced with independence of this genetic factor.

Other interesting aspect of our MASEI results is that only patients older than 50 years of age showed values over the threshold identifying patients with AS or SpA [31], [33]. Therefore, our results do not question the specificity of this threshold for early SpA, which starts most often below this age. An increase of enthesal abnormalities with age was already shown more than a decade ago [46]. It was described as independent of the underlying disease and, as in our study, to be mostly due to calcification.

Two of the other clinical features we have analyzed were different between HLA-B27 positive and negative patients with RA. The first was the prevalence of RF, which was less common in HLA-B27 positive patients. It was accompanied by a trend to lower prevalence of ACPA. This result could imply either association of HLA-B27 with RF seronegative RA or misclassification of patients. The latter possibility can be reasonably excluded because we only found a patient with SpA among the 41 that were specifically revised. In addition, the HLA-B27+ RF- patients were not different in any respect from the HLA-B27- RF- subgroup. For example, the HLA-B27+RF- and B27-RF- patients were comparable at percentage of ACPA+, 26.7 % vs. 25.6 %, erosive arthritis, 51.7 % vs. 45.7 %, or percentage of carriers of SE, 44.4 % vs. 43.1 %, respectively. This leaves us with the possibility that HLA-B27 could be a susceptibility factor for RF seronegative RA. Only studies in additional sample collections will be needed to clarify this matter.

An increased prevalence of low back pain was also associated with HLA-B27 in the patients with RA. This outcome from the anamnesis was combined with the identification of two patients with radiographic sacroiliitis among the patients with inflammatory low back pain. The two results are in agreement with the idea that HLA-B27 could modify RA towards the axial joints. This idea is supported by previous studies showing association between sacroiliitis and HLA-B27, both in HLA-27-associated diseases [17], [19], [20], [21], and in some non-associated diseases [22], [23], [24], [26] including RA [10], [12], [13]. In fact, this is the unique association with HLA-B27 that has been replicated in different RA studies. However, two previous studies did not found differences in the sacroiliac joints between HLA-B27 positive and negative patients with RA [47], [48]. These findings should motivate new studies directed to the analysis of low back pain and to the identification of sacroiliitis. MRI will be the technology of choice for these studies because it is very sensitive for incipient changes in the sacroiliac joints [29], and it could provide more definitive results than radiography given the small size of the HLA-B27 positive subgroup.

In relation with the low back pain association, it could be argued that its presence questions the classification of the patients as RA. However, this type of reasoning is incompatible with the aim of our study. When the aim is to identify patients with RA showing phenotypes resembling SpA, we need to consider classification of RA before doing any extra analysis and keep this classification constant along the study.

## Conclusions

We have found that HLA-B27 positive patients with RA do not have more enthesitis as assessed with US than those lacking this allele. However, these patients referred a more prevalent story of low back pain and were more often seronegative for RF than the HLA-B27 negative patients indicating that HLA-B27 could be shaping the RA phenotype in other directions deserving further and more focused analysis.
